# One-step nondestructive functionalization of graphene oxide paper with amines†

**DOI:** 10.1039/c8ra00986d

**Published:** 2018-04-23

**Authors:** Natalia Alzate-Carvajal, Diego A. Acevedo-Guzmán, Victor Meza-Laguna, Mario H. Farías, Luis A. Pérez-Rey, Edgar Abarca-Morales, Victor A. García-Ramírez, Vladimir A. Basiuk, Elena V. Basiuk

**Affiliations:** Centro de Ciencias Aplicadas y Desarrollo Tecnológico, Universidad Nacional Autónoma de México, Circuito Exterior C.U. Ciudad de México 04510 Mexico elbg1111@gmail.com; Instituto de Ciencias Nucleares, Universidad Nacional Autónoma de México, Circuito Exterior C.U. Ciudad de México 04510 Mexico basiuk@nucleares.unam.mx; Centro de Nanociencias y Nanotecnología, Universidad Nacional Autónoma de México Apdo. Postal 14 Ensenada 22800 Baja California Mexico

## Abstract

Direct functionalization of prefabricated free-standing graphene oxide paper (GOP) is the only approach suitable for systematic tuning of its mechanical, thermal and electronic characteristics. However, the traditional liquid-phase functionalization can compromise physical integrity of the paper-like material up to its total disintegration. In the present paper, we attempted to apply an alternative, solvent-free strategy for facile and nondestructive functionalization of GOP with 1-octadecylamine (ODA) and 1,12-diaminododecane (DAD) as representatives of aliphatic amines, and with 1-aminopyrene (AP) and 1,5-diaminonaphthalene (DAN) as examples of aromatic amines. The functionalization can be carried out under moderate heating at 150–180 °C for 2 h in vacuum, and proceeds through both amidation and epoxy ring opening reactions. Comparative characterization of pristine and amine-modified GOP samples was carried out by means of Fourier-transform infrared, Raman, and X-ray photoelectron spectroscopy, thermogravimetric and differential thermal analysis, scanning electron and atomic force microscopy. In addition, we compared stability in water, wettability, electrical conductivity and elastic (Young's) modulus of GOP samples before and after functionalization. The highest content of amine species was obtained in the case of GOP-ODA, followed by GOP-DAD, GOP-AP and GOP-DAN. The functionalization increased mechanical and thermal stability, as well as the electrical conductivity of GOP. The magnitude of each effect depends on the structure of amine employed, which allows for tuning a given GOP characteristic. Morphological characterization showed that, compared to pristine graphene oxide paper, amine-modified mats become relatively ordered layered structures, in which individual GO sheets are organized in a near-parallel fashion.

## Abstract 2

Solvent-free functionalization of graphene oxide paper with amines is facile, nondestructive, and attractive from economic and environmental points of view.

## Graphical abstract

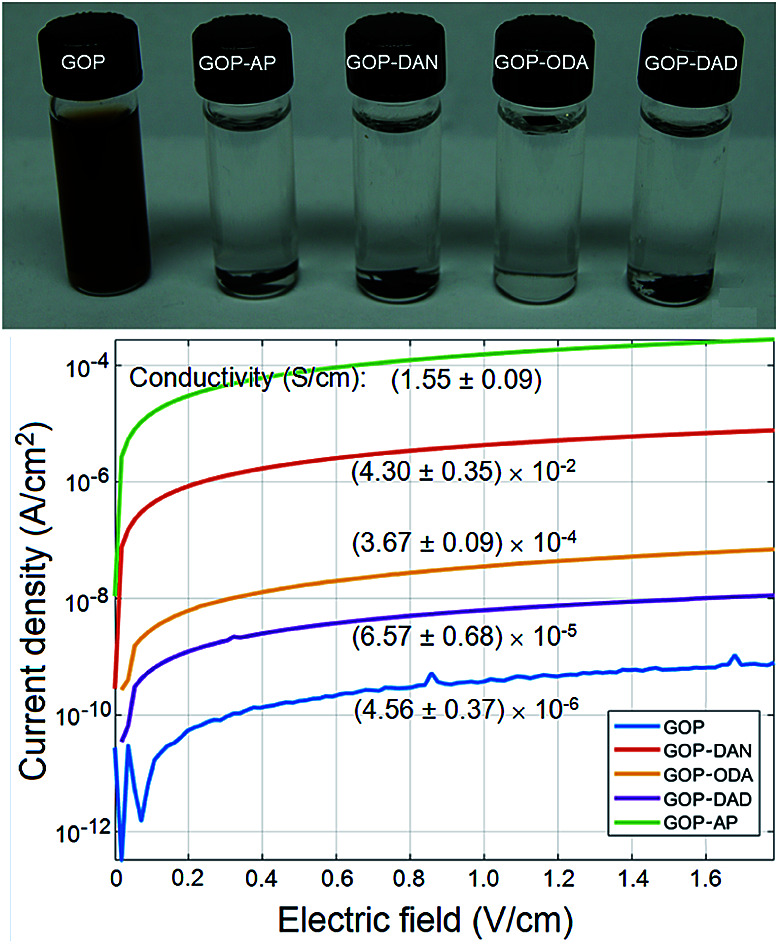

## 1. Introduction

The necessity in the development of layered nanostructures with tunable properties has brought close attention to graphene oxide (GO) paper (GOP) as a very promising material for such diverse application as the fabrication of supercapacitors, fuel cells, Li-ion batteries, chemical and biochemical sensors, UV-visible photosensors, flexible surface-enhanced Raman scattering substrates, magnetic and thermal conductive materials, nanofiltration membranes, and bactericidal agents for water disinfection, among others.^1^ GOP is a layered material composed of stacked oxidized graphene sheets with a variety of oxygen-containing functionalities such as epoxy and hydroxyl groups (mainly on basal plane) and carbonyl and carboxyl groups distributed throughout the sheet edges.^2–8^ Such a rich surface chemistry can serve as a starting point for successful chemical (covalent, noncovalent, ionic and coordination) functionalization.^4,5^ The main obstacle for the use of GOP in certain applications is that it is an insulating material due to the disrupted sp^2^ bonding network.^9^ A number of research reports have demonstrated that thermal and chemical treatments could enhance the electrical conductivity of GOP, however not without concerns that such treatments can affect its integrity. The reduction with hydrazine is an example of the most common methods to improve electronic as well as mechanical characteristics of GOP.^10^ Another type of processes currently used for the same purposes is thermal annealing. This treatment is usually carried out at temperatures between 300 and 500 °C, and can increase GOP conductivity up to 200 S cm^−1^.^11^ Furthermore, as reported by Vallés *et al.*,^12^ annealing GOP at temperatures of about 700 °C under argon atmosphere can result in even higher conductivity values, of up to 8000 S cm^−1^. Unfortunately, both methods degrade severely the chemical structure of GO by removing an important amount of oxygen-containing functionalities and generating graphene structure in this way.

A suitable alternative pathway to enhance the electrical conductivity of GOP without harsh alterations of the surface chemistry of graphene oxide sheets is chemical functionalization with organic molecules. For this purpose, the same techniques as those usually employed for functionalization of GO powder can be employed.^13–15^ In general terms, the preparation of functionalized GOP can be performed by following two different strategies.^15*b*,*c*^ The first one consists in chemical treatment of graphene oxide sheets of the powder prior to the assembly of free-standing paper-like material, which requires that the chemically modified nanoplatelets are well dispersible in a solvent prior to filtration. The alternative strategy implies that chemical modification is carried out after the formation of GOP, where the most crucial requirement is that functionalizing molecules must be capable of diffusing into the inter-platelet spaces in order to further react with oxygen-containing functional groups.

Only the second approach is suitable for systematic chemical modification of GO papers,^15*b*,*c*^ allowing for tuning of their electronic and mechanical characteristics. Unfortunately, at present, it remains underexplored. The main underlying problem is that the traditional liquid-phase functionalization can compromise physical integrity of the paper-like material up to its total disintegration. However, it turns out that the liquid-phase approach is not the only possible one to be employed for GOP functionalization. A promising alternative approach which, in principle, can help to avoid the negative effects of a solvent medium is the solvent-free functionalization, which is successfully applied by our research group for both covalent^16,17^ and noncovalent^18^ attachment of different chemical species, mainly amines,^16,17^ to carbon nanomaterials, comprising carbon nanotubes of different types, nanodiamond, as well as GO powder. The possibility of avoiding the use of solvents has additional ecological and economic implications, since the wastes and necessary equipment are reduced to a minimum: in particular, no ultrasonication, filtration and centrifugation systems are required. The time needed to complete functionalization and obtain the target material is also dramatically reduced to a few hours only.

**Fig. 1:**
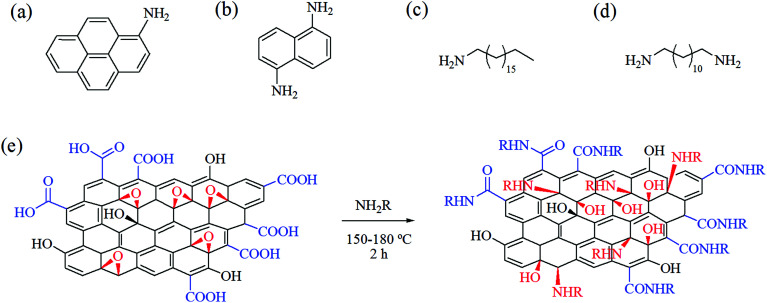
Chemical structures of amines employed in the present study: (a) 1-aminopyrene, (b) 1,5-diaminonaphthalene, (c) 1-octadecylamine and (d) 1,12-diaminododecane. (e) Suggested general scheme for the transformation of GOP functional groups (carboxylic, in blue; epoxy, in red) upon functionalization with amines. R = aryl or alkyl.

Bearing in mind the above advantages, in the present work we attempted to apply the solvent-free strategy for facile and nondestructive functionalization of GOP with four amines of variable structure (Fig. 1a–d). 1-Octadecylamine (ODA) and 1,12-diaminododecane (DAD) were representatives of aliphatic amines, whereas 1-aminopyrene (AP) and 1,5-diaminonaphthalene (DAN), examples of aromatic amines. Two of the amines employed were monofunctional (ODA and AP), and another two, diamines (DAD and DAN). Comparative characterization of non-functionalized and amine-modified GOP samples was carried out by means of Fourier-transform infrared (FTIR), Raman and X-ray photoelectron spectroscopy (XPS), thermogravimetric and differential thermal analysis (TGA and DTA, respectively), scanning electron microscopy (SEM), and atomic force microscopy (AFM). In addition, we compared stability in water, wettability, electrical conductivity and elastic (Young's) modulus of GOP samples before and after amine functionalization.

## 2. Experimental

### 2.1. Materials

GO powder (>99 wt% purity, platelet diameter of 0.5–3 μm and thickness of 0.55–1.2 nm) from Nanostructured and Amorphous Materials, Inc., was used. 1-Octadecylamine (97%), 1,12-diaminododecane (98%), 1-aminopyrene (97%) and 1,5-diaminonaphthalene (97%) from Sigma-Aldrich were used as received. All of them are thermally stable compounds and sublime under moderate vacuum at temperatures below 200 °C.

### 2.2. Preparation of graphene oxide paper

Aqueous dispersions of graphene oxide powder were obtained by ultrasonic treatment, where 40 mg of GO was added to 6 mL of water. The dispersions were filtered through cellulose acetate membrane filter (0.45 μm pore size, Whatman), using a conventional Millipore filtration system. The mats formed on the filter were dried in a vacuum desiccator. Finally, the resulting GOP samples were mechanically removed from the cellulose filter. The free-standing mats obtained in this way are hereafter referred to as pristine GOP.

### 2.3. GOP functionalization

Prior to functionalization, GOP samples were degassed for 1 h at *ca.* 100 °C under constant evacuation at about 10^−2^ torr. The treatment with amines was performed in a Pyrex glass reactor, in which GOP was placed together with amine reagent at GOP : amine w/w ratio of 1 : 5 and heated for 2 h. Other conditions depended on a particular amine: the functionalization with AP and DAN was performed at 175–180 °C with intermittent evacuation, and the treatment with ODA and DAD, at 150 °C under static vacuum. After the above procedure, in all cases, the reactor was additionally heated at about 150 °C under constant vacuum for 1 h to remove the excess of amines. The scheme suggested for the reactions of GOP with amines is presented in Fig. 1e: it contemplates the possibility of formation of amide derivatives with carboxylic groups of GO sheet edges, and amine addition onto epoxy groups of basal planes. The functionalized samples prepared are denoted hereafter as GOP-AP, GOP-DAN, GOP-ODA and GOP-DAD.

### 2.4. Characterization

FTIR spectra were acquired using a Nexus 670 FTIR Thermo-Scientific Nicolet iS50R instrument, under room temperature and atmospheric pressure. Raman spectra were recorded on a Thermo-Nicolet Almega Dispersive Raman Instrument (*λ* = 532 nm).

For XPS studies, we employed a SPECS GmbH custom made X-ray photoelectron spectrometer microprobe, equipped with a PHOIBOS 150 WAL hemispherical analyzer and a monochromated Al Kα X-ray source (μ-FOCUS 500) with an energy of 1486.6 eV. XPS survey spectra were acquired for an extensive binding energy range with a 1 eV step size, while high-resolution energy regions with a range of 30 eV were designated for all elements of interest (C 1s, N 1s, and O 1s) using a 0.1 eV step size and a dwell time of 0.2 s. Spectra are presented without smoothing. Charge referencing was done against adventitious carbon by setting the C 1s peak maximum at 284.7 eV.

TGA–DTA curves were acquired by using an SDT-Q600 analyzer from TA Instruments, under an air flow of 100 mL min^−1^ and with a heating ramp of 10 °C min^−1^. For SEM characterization of GOP mats, we employed a JEOL JSM-6510LV instrument functioning in low voltage mode at 5 kV. AFM images were obtained using a JEOL JSPM-5200 instrument in tapping mode, for the samples adhered to silicon wafers.

Wettability of GOP sample surfaces with water was analyzed by measuring the contact angle, using the Drop Shape Analyzer DSA25 from KR  ŰSS GmbH. The surface free energy was calculated by using the Owens–Wendt–Rabel–Kaelble (OWRK) model,^19^ in which the surface free energy is divided into polar and disperse part. The relationship (commonly referred to as Young's equation) between the contact angle *θ*, the surface tension of the liquid *γ*_l_, the interfacial tension *γ*_sl_ between liquid and solid and the surface free energy *γ*_s_ of the solid is as follows:

1 *γ*_s_ = *γ*_sl_ + *γ*_l_ cos(*θ*)

The interfacial tension *γ*_sl_ is calculated based on the surface tensions *γ*_s_ and *γ*_l_ between the phases. These interactions are interpreted as the geometric mean of disperse part *γ*^D^ and polar part *γ*^P^ of the surface tension or surface free energy:2
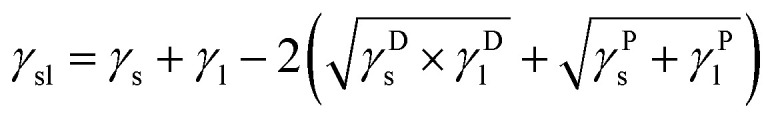


At least two liquids (we used water and diiodomethane as reference) with known disperse and polar parts of the surface tension are required to determine the surface free energy of the solid, wherein at least one of the liquids must have polar part >0. Further details are specified in the ESI.†

The Young's modulus of GOP samples was evaluated on a home-made device, based on the cantilever beam principles similar to those described elsewhere.^20^

Conductivity measurements were carried out under ambient conditions using a Keithley 2601B Source Meter Unit (SMU) operating in a DC mode and controlled *via* a PC. The GOP mats were cut into strips of 5 mm and placed perpendicularly to parallel copper electrodes. The dimensions of the electrodes, printed on a circuit board, were of 0.25 mm in width and separated by 2.8 mm. Electric contact was obtained by lightly pressing a piece of glass slide upon the printing board by a clip system. The samples were maintained in a vacuum desiccator for 24 h before each measurement. The SMU was programmed to measure the current in the sample after applying several voltages within a given range. The procedure for measuring the current consisted of the following steps. First, a positive voltage supply (+Δ*V*) was applied to the sample and the electrical current was measured. Second, a voltage supply of the opposite polarity (−Δ*V*) was applied to the sample to eliminate possible effects of the background currents produced by the +Δ*V* voltage applied previously. Third, these two steps were repeated after increasing the voltage by a *δV* value until the final voltage (*V*_f_) was reached. The voltage *δV* was chosen in such a way that one hundred points were measured. This method, known as alternating polarity, is used to improve the measurement quality for high-resistivity samples,^21^ which are prone to produce large errors due to background currents, as it occurs in the case of GO. We applied this protocol for all the samples with the purpose of obtaining comparable measurements for pristine and functionalized GOP. The measurements were repeated at least 5 times for each sample showing a good reproducibility.

## 3. Results and discussion

**Fig. 2:**
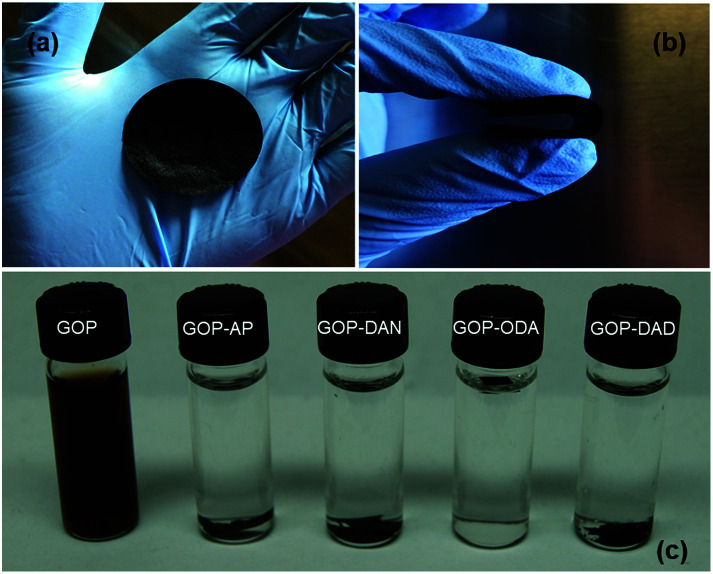
GOP samples obtained (a) which can be folded without breaking (b). (c) Dispersibility test for pristine and amine-functionalized GOP samples in water after 10 min sonication. After three months, the functionalized samples remained without visible changes.

Filtration of aqueous GO dispersions results in a paper-like homogeneous material. GOP obtained (Fig. 2a and b) is a flexible and easy to handle material with a slight metallic shine on its surface. The paper can be folded and rolled without breaking or fracturing, suggesting that the individual GO sheets within the paper form uninterrupted networks, which provides the paper material high structural and mechanical stability. The mat thickness as determined by SEM can vary in the range of 16–30 μm (see below).

A dispersibility test (Fig. 2c) was employed as a simple evidence of changes in the chemical nature of GO surface due to amine functionalization. Only pristine GOP mats disintegrated completely after ultrasonic bath treatment for 10 min, resulting in a brown solution, similar to the solutions formed by graphene oxide powder.^22^ The introduction of amine moieties into GOP enhances dramatically stability of the samples in water. Despite of the structure of all four amines employed is distinct (two aromatic and two aliphatic amines), all functionalized GOP mats after ultrasonication remain visually intact even after three months, demonstrating that the introduction of amine functionalities gives rise to water resistant GOP. This can be explained by the introduction of highly hydrophobic functionalities into graphene oxide structure, which are long hydrocarbon chains in the case of GOP-ODA and GOP-DAD, and aromatic rings for GOP-AP and GOP-DAN samples. In the former case, aliphatic chains are responsible for strong hydrophobic interactions between individual graphene oxide sheets, and in the latter case, the stabilizing effect is due to π–π stacking.

**Fig. 3:**
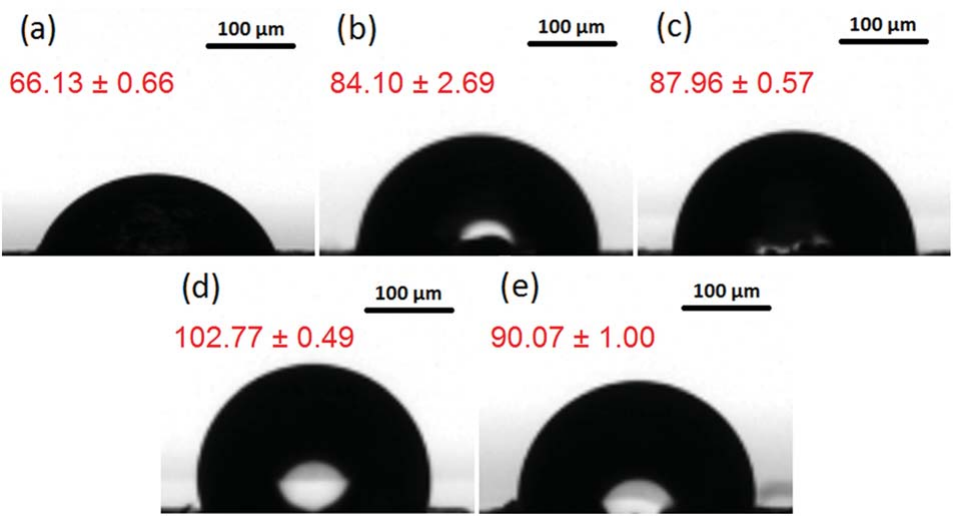
Images for contact angle (values in red) measurements for: (a) pristine GOP, (b) GOP-AP, (c) GOP-DAN, (d) GOP-ODA and (e) GOP-DAD.

The comparative wettability of GOP surfaces was characterized by measuring the contact angle of the samples before and after functionalization with amines (Fig. 3). From the contact angles obtained, the values of surface free energy were calculated, which are specified in Table 1. Pristine GOP exhibited the lowest value of contact angle due to the presence of hydrophilic oxygen-containing groups on the surface of individual graphene oxide sheets. The grafting of aromatic and aliphatic amines onto the latter increased the water contact angle as well as decreased the surface free energy, thus indicating significant changes in intermolecular interactions between a liquid (water) and a solid (GOP) after functionalization. This effect is more pronounced for aliphatic amines then for aromatic ones, furthermore, it becomes more evident with increasing the alkyl chain length. In particular, as one can see from Fig. 3 and Table 1, the least wettable GOP was obtained after functionalization with ODA, resulting in the highest contact angle of 102.77°, and the lowest value for the surface free energy of 24.55 mJ m^−2^.

**Table 1:** Contact angle and surface free energy values for pristine and functionalized GOP samples

Sample	Contact angle (°)	Surface free energy (mJ m^−2^)
GOP	66.13 ± 0.66	48.43 ± 0.50
GOP-AP	84.10 ± 2.69	41.18 ± 3.22
GOP-DAN	87.96 ± 0.57	40.05 ± 0.74
GOP-ODA	102.77 ± 0.49	24.55 ± 0.24
GOP-DAD	90.07 ± 1.00	42.18 ± 0.29

The changes in mechanical stability of GOP samples after gas-phase treatment with amines were evaluated by applying the cantilever test based on optical measurements, working on similar principles to those described in ref. 20. For this purpose, a home-made device was assembled and tested, using for calibration the values of Young's modulus reported elsewhere for nylon^23^ and pristine GOP.^4,8^ The measurements of Young's modulus for our pristine GOP samples gave a value of 13.6093 GPa (Table 2), which is in a good accordance with the value of 16.6 GPa reported by another research group.^4^ We did not find dramatic changes in Young's modulus for GOP-DAD, GOP-AP, and GOP-DAN. However, in the case of GOP-ODA, a very high modulus value of 256.19 GPa was obtained. In line with the results of wettability tests, this observation can be attributed to the fact that amine molecules with the longest alkyl chain are capable of generating most considerable changes in the structure and mechanical stability of GOP; a detailed mechanistic explanation is based on the changes in GOP microstructure, which will be discussed below (within the results of SEM imaging).

**Table 2:** Values of Young's modulus for pristine and functionalized GOP samples. For comparison, the values for nylon and pristine GOP reported elsewhere are specified in parenthesis

Material	Young's modulus (GPa)	Correlation coefficient
Nylon	1.4869 (1.4 (ref. 22))	0.9997
GOP	13.6093 (16.6 (ref. 4), 32 (ref. 8))	0.9998
GOP-AP	16.7787	0.9897
GOP-DAN	17.9368	0.9967
GOP-ODA	256.1856	0.9867
GOP-DAD	21.8667	0.9945

**Fig. 4:**
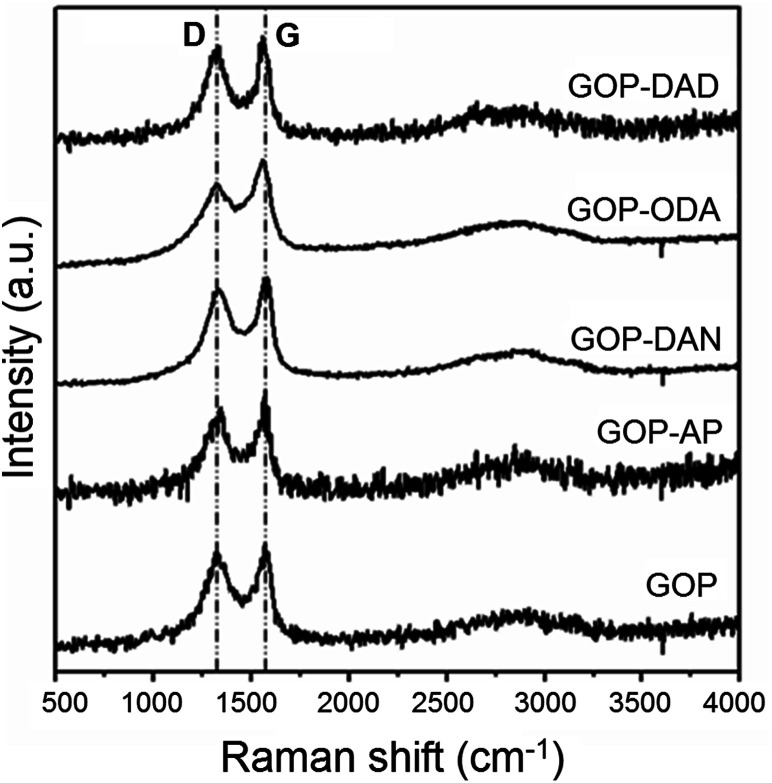
Raman spectra of GOP before and after functionalization with amines.

Several spectroscopic methods were employed to characterize the changes in chemical nature of GO sheets due to amine functionalization. Raman spectra of all the samples (Fig. 4) show the presence of characteristic D-band at 1338 cm^−1^ associated with the disorder induced in graphene sheets, and graphene G band (at 1565 cm^−1^ for pristine GOP) due to the first order scattering E_2g_ mode.^24^ The ratio between the intensity of D and G Bands (*I*_D_/*I*_G_) did not show substantial changes and decreased from 1.39 for pristine GOP to 1.30 for all amine-functionalized samples. Since the intended functionalization route relied upon reactions with oxygen-containing functionalities of GO, and not with the graphite basal plane composed of sp^2^-hybridized carbon atoms, no variations in *I*_D_/*I*_G_ intensity ratio were expected. The only observation worth of mentioning is a slight G-band shift from 1565 to 1578 and 1576 cm^−1^ for GOP-AP and GOP-DAN, respectively, which is apparently caused by the contribution of sp^2^ carbon atoms of aromatic amine molecules.^25^

**Fig. 5:**
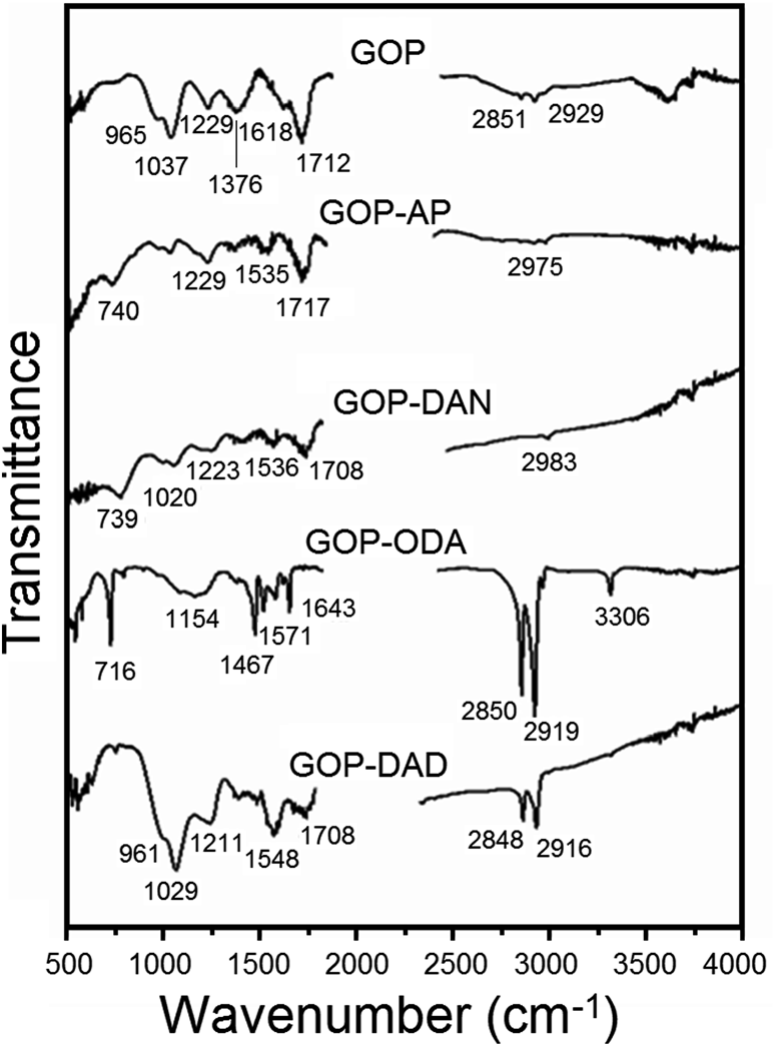
FTIR spectra of pristine and functionalized GOP samples.

On the other hand, FTIR analysis turned to be more informative and detected evident changes associated with the chemical modification of GO surface (Fig. 5). Pristine GOP exhibits typical peaks due to numerous oxygen-containing functional groups usually existing on individual sheets of graphene oxide. In addition to a broad hydroxyl *ν*_OH_ band at about 3600 cm^−1^ and the corresponding *δ*_OH_ band at 1618 cm^−1^, one can observe a peak at 1229 cm^−1^ due to O–H deformation mode of C–OH groups and a broad band at 1037 cm^−1^, attributed to C–O stretching vibrations.^26^ Also, the same spectrum shows a characteristic feature of GO at 1712 cm^−1^, corresponding to *ν*_C=O_ vibrations in COOH groups, likewise a strong band at 1376 cm^−1^ due to epoxy C–O–C bonds,^26*b*^ a shoulder at 965 cm^−1^ due to unsaturated ketone groups, as well as very weak symmetric and asymmetric *ν*_C–H_ bands at 2851 and 2929 cm^−1^. The most obvious changes observed after amine treatment are an almost total disappearance of the epoxy feature, a considerable decrease in the intensity of *ν*_C=O_ band (up to its total disappearance in the case of GOP-ODA) and of the C–O signal at 1037 cm^−1^. On the other hand, new bands appeared between 1535 and 1571 cm^−1^, which are commonly associated with *δ*_NH_ vibrations, including those found in amide group.^27^ These spectral features are similar to the ones observed for amine functionalization of GO powders,^16*j*^ where amines molecules are attached to graphene oxide surface not only through amidation of COOH groups, but also through ring-opening reaction of epoxy groups. For GOP-ODA, a clear evidence of amide bond formation is the appearance of “amide I” (*ν*_C=O_) band at 1643 cm^−1^ and “amide II” (*δ*_NH_) band at 1571 cm^−1^; the signals at 716, 1154, 1467 and 3306 cm^−1^ can be attributed to N–H wagging, *ν*_C–N_, *δ*_CH_ and *ν*_NH_ vibrations (in secondary NH moieties formed as a result of ODA addition to epoxy groups), respectively. For GOP-AP and GOP-DAN, the band located at about 740 cm^−1^ is associated with C–H wagging vibrations in the aromatic rings; weak aromatic *ν*_C–H_ absorption can be found at 2975 and 2983 cm^−1^, respectively. For GOP-ODA and GOP-DAD, which were functionalized with aliphatic amines, the additional bright spectral features are sharp bands at 2848–2850 and 2916–2919 cm^−1^ due to CH stretching vibrations in long aliphatic hydrocarbon chains. For GOP-DAD, additional well-manifested features are the ones at 961, 1029 and 1211 cm^−1^ due to unsaturated ketone groups, C–O stretching vibrations and O–H deformation mode of C–OH groups, respectively.

**Fig. 6:**
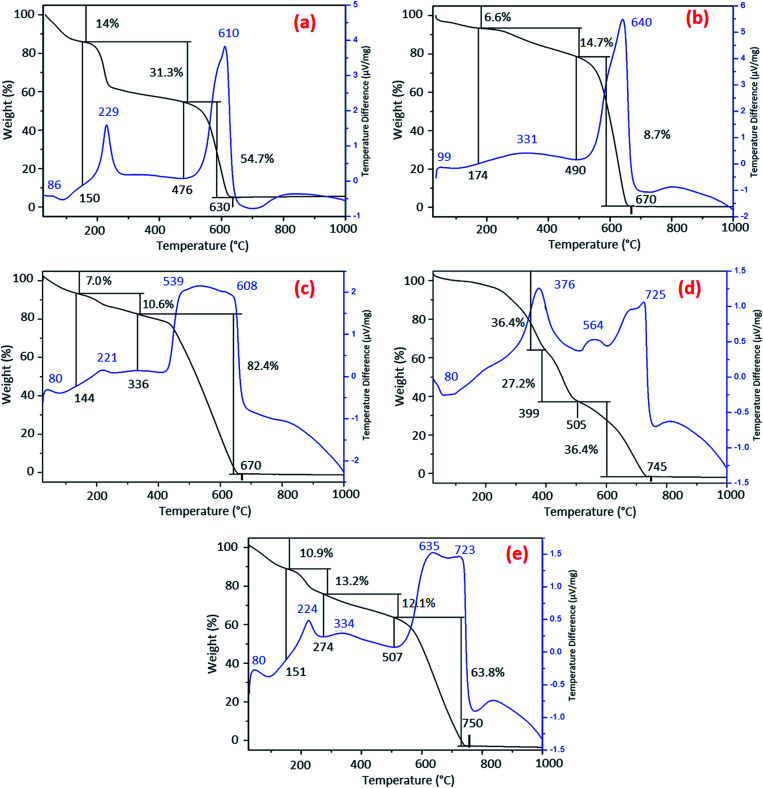
TGA-DTA curves for (a) pristine GOP, (b) GOP-AP, (c) GOP-DAN, (d) GOP-ODA and (e) GOP-DAD.

TGA–DTA measurements were undertaken in order to explore the changes in the thermal behavior of GOP samples after amine treatment, as well as to estimate an approximate degree of chemical functionalization. The TGA curve for pristine GOP (Fig. 6a) is quite typical for graphene oxide, exhibiting three weight loss steps.^28,29^ The initial weight loss of 14% occurred until about 150 °C is due to evaporation of physisorbed water, which is always present on GO surfaces. The second weight loss of 31.3%, observed until 476 °C, is caused by the removal of intrinsic oxygen-containing groups of GO. The third and final weight loss of 54.7%, ending at 630 °C, corresponds to the decomposition of graphene lattice. The DTA curve for pristine GOP has three exothermic peaks around 86, 229 and 610 °C, which is consistent with the three weight loss steps found in TGA curve.

The TGA curves obtained for amine-treated GOP samples exhibit considerable differences as compared to the one of pristine GOP. The thermogram for GOP-AP (Fig. 6b) has three main weight losses of 6.6% until 174 °C, 14.7% until 490 °C and 78.7% until 670 °C. The DTA curve showed one weak exothermic peak at 99 °C followed by two major peaks at 331 °C and 640 °C, matching the number of weight losses found by TGA. The first step is associated with the elimination of adsorbed water; the second step is related to the oxidation of covalently (and probably some noncovalently) bonded AP molecules; and the final step is due to the decomposition of graphene backbone. For GOP-DAN sample (Fig. 6c), the first weight loss of 7.0% was observed until 144 °C, the second one of 10.6% until 336 °C, and the final loss of 82.4% until 670 °C. The corresponding DTA curve showed a small exothermic peak at 80 °C due to the removal of physisorbed water, followed by a peak at 221 °C, most likely due to the oxidation of a small amount of noncovalently bonded DAN molecules, and by a high and broad exothermic peak at 539 °C with a shoulder at 608 °C, which can be associated with the oxidation of covalently attached DAN moieties followed by the decomposition of graphene network. In the case of functionalization with aliphatic amine ODA (Fig. 6d), the initial weight loss due to adsorbed water is very insignificant, which goes in line with the highest hydrophobicity of this sample. The three most important weight losses in TGA curve are those of 36.4% until 399 °C, of 27.2% until 505 °C, and of 36.4% until 745 °C, which is much higher final decomposition temperature than that for pristine GOP, GOP-AP and GOP-DAN. The most important exothermic peaks in DTA curve for GOP-ODA are found at 376, 564 and 725 °C. The latter peak can definitely be attributed to the final decomposition of graphene network. The former two peaks must be related to the oxidation of different ODA species: some of them can be amide species resulting from condensation of ODA with COOH groups, others, from the addition onto epoxy groups, with a possible contribution of noncovalently bonded amine molecules. In the case of functionalization with a bifunctional aliphatic amine, DAD, the TGA curve (Fig. 6e) looks rather similar to the one obtained for GOP-DAN (Fig. 6c), derived from another bifunctional, but aromatic amine. The main weight losses observed are as follows: of 10.9% until 151 °C, of 13.2% until 274 °C, of 12.1% until 507 °C, and the final weight loss of 63.8% until 750 °C, which is, again, much higher final decomposition temperature than that for pristine GOP, GOP-AP and GOP-DAN. The DTA curve for this sample showed five exothermic peaks. The first peak at 80 °C corresponds to the removal of adsorbed water; the second peak at 224 °C can be explained by the presence of noncovalently bonded amine species; the third peaks at 334 °C is associated with the decomposition of covalently attached DAD molecules. The remaining two are high-temperature peaks at 635 and 723 °C. Here one should emphasize that DAD is a long chain aliphatic diamine, which offers two general bonding possibilities, by involving only one or both NH_2_ groups: the latter case can result in cross-linking of adjacent GO sheets. Because of the high temperatures, the fourth and fifth exothermic peaks both correspond to the final oxidation of graphene sheets, but the one at 635 °C is due to burning individual sheets, whereas the highest-temperature peak at 723 °C can result from the decomposition of cross-linked graphene sheets. Because of the qualitative similarity of TGA/DTA data for GOP-DAD and GOP-DAN, one can suggest that the cross-linking phenomena can take place in the case of aromatic DAN as well; however, the contribution of cross-linking in the latter case is less significant due to a smaller size of DAN moieties.

The high-temperature DTA peak at 725 °C, as well as the corresponding high-temperature weight loss ending at 750 °C, observed in the case of GOP-ODA, at first glance is more difficult to explain. Due to the monofunctionality of ODA, covalent cross-linking is impossible. Nevertheless, very long aliphatic chains of ODA, attached to adjacent GO sheets, are known to bind very strongly to each other through van der Waals interactions, due to which pillar-supported frameworks can form,^30^ whose stability is comparable with that of covalently cross-linked GO frameworks.

Thus, all amine-modified GOP samples exhibit a higher thermal stability than the one found for pristine GOP (total decomposition at 630 °C): complete combustion is observed at 670, 670, 745 and 750 °C for GOP-AP, GOP-DAN, GOP-ODA and GOP-DAD, respectively. This increase in thermal stability of graphene oxide backbone is qualitatively similar to that observed for amine-functionalized GO powders.^16*j*^

From the analysis of weight losses in TGA curves due to the decomposition of graphene backbone *versus* all other structural elements including organics, one can suggest that the highest content of amine species is observed in GOP-ODA (the lowest weight loss of 36.4% due to graphene), followed by GOP-DAD (63.8%), GOP-AP (78.7%) and GOP-DAN (82.4%). More precise estimates of the degree of functionalization are impossible, since the lower-temperature weight losses account not only for amine species, but also for the decomposition of oxygen-containing groups, which did not react with amines.

**Fig. 7:**
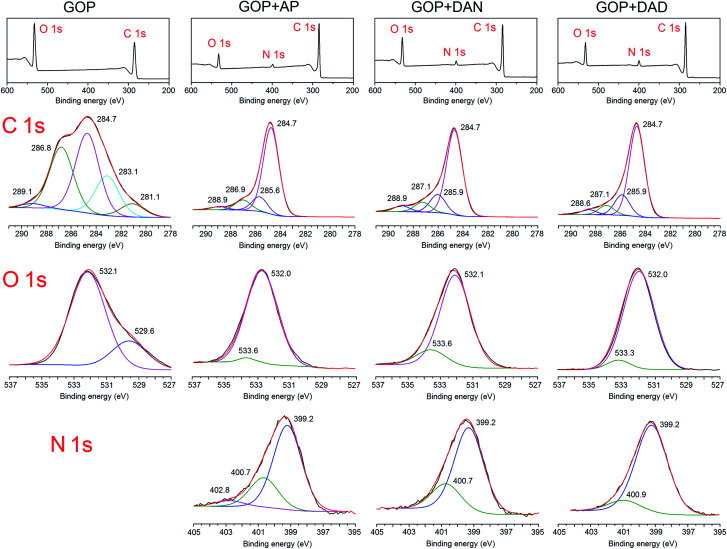
Comparison of XPS spectra for GOP samples before and after functionalization with amines: survey spectra (top row) along with deconvolution of the corresponding C 1s, N 1s and O 1s signals. Raw spectra are shown in black, and sum, in red.

A more detailed analysis of the changes in chemical nature of GOP after the gas-phase treatment with amines was undertaken by using XPS technique. The survey spectra (Fig. 7, top row) show the presence of C 1s and O 1s peaks for both pristine and functionalized GOP samples, and the appearance of a clear N 1s signal around 400 eV for all amine-treated samples. The deconvolution of C 1s peak for pristine GOP revealed the presence of the well-known components at binding energies of 284.7 (sp^2^/sp^3^ C–C bonds), 286.8 (epoxy groups) and 289.1 eV (O–C=O moieties).^31,32^ The presence of lower-energy components below 284 eV is less common (we observed it for GOP only, and not for amine treated mats); other authors^33,34^ attributed them to the presence of carbon vacancies in GO sheets, with particular binding energy values of 283.4 (ref. 33) and 283.6 (ref. 34) eV. The appearance of a new component at 288.6–288.9 eV (–N–C=O),^35^ decrease of the peak due to epoxy groups, an almost total disappearance of the carboxyl O–C=O component, as well as the appearance of a new component at 285.6–285.9 eV (usually associated with C–N bond in amine moieties attached to epoxy groups) suggests that amine molecules were grafted to graphene oxide sheets in GOP, as it was expected, through both amidation reaction and amine addition onto epoxy rings. The deconvolution of N 1s core-level signals supports the above interpretation. The spectra of all functionalized GOP samples have the component with a binding energy of 400.7–400.9 eV, assigned to protonated amine/amide species, and a major peak at 399.2 eV corresponding to the secondary amino groups resulting from the addition onto epoxy rings.^16*j*,36^

As regards the oxygen-related components, we prefer to limit ourselves to the most general interpretation only, since many assignments for the “carbon–oxygen” groups offered in the literature are conflicting, for different reasons.^35^ The first factor to be mentioned is that O 1s photoelectron kinetic energies are lower than those for C 1s, the O 1s sampling depth is smaller, and correspondingly the O 1s spectra are more surface specific. The second reason is a broad variety of oxygen-containing functionalities existing in GO sheets, which can contribute to the O 1s peak. An additional factor is the possibility of contamination with atmospheric oxygen. One can say for sure that the intense peak at 532.1 eV for pristine GOP comprises the most characteristic bonds including C=O and C–O in carboxylic and epoxy groups.^35,37^ The peak at 529.6 eV can be due to quinones. For all amine-treated GOP samples, the latter feature disappears completely. On the other hand, an important component found after functionalization is the one at 533.3–533.6 eV, which can be assigned to hydroxyl groups resulting from epoxy ring opening. The component corresponding to O atoms in amide moieties would fall into the region of 531–533 eV, most likely very close to the peak position for carboxylic oxygen. As a whole, XPS results, along with FTIR and TGA/DTA data obtained, provided strong evidence for the formation of covalent bonds during the gas-phase functionalization of GOP with amines.

**Fig. 8:**
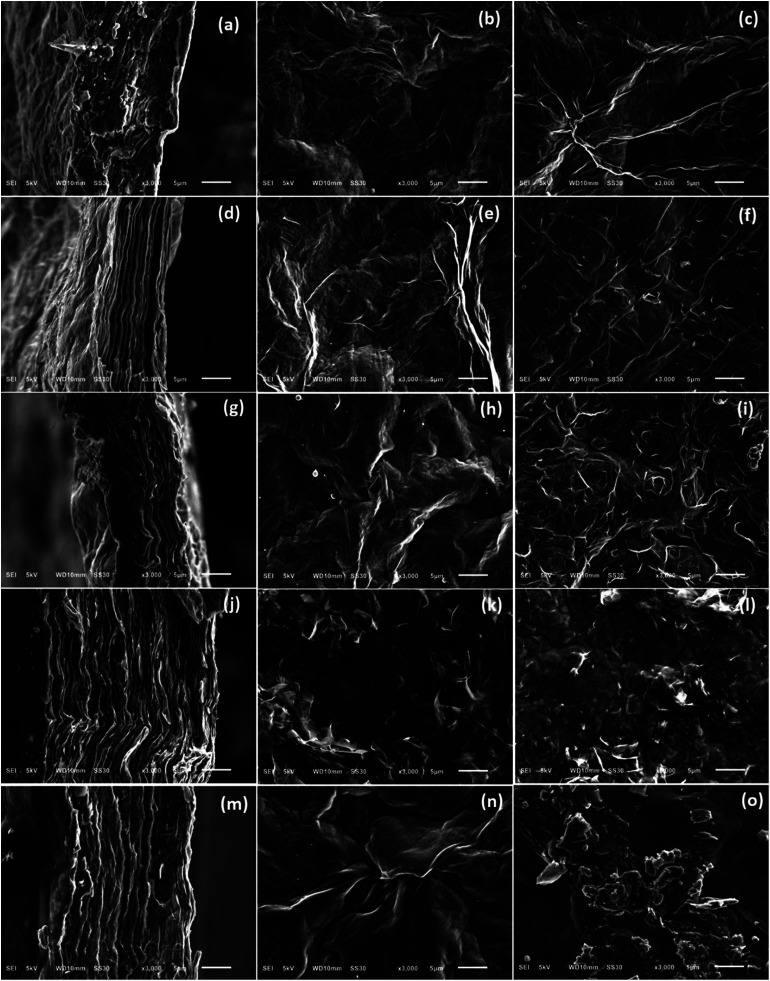
Representative SEM images of pristine GOP (a–c), GOP-AP (d–f), GOP-DAN (g–i), GO-ODA (j–l) and GOP-DAD (m–o). The left column (a, d, g, j, m) shows cross-section images; the middle column (b, e, h, k, n) corresponds to the upper mat side; the right column (c, f, i, l, o), to the lower mat side which was in contact with the filter membrane during GOP fabrication. Scale bar, 5 μm in all images.

**Fig. 9:**
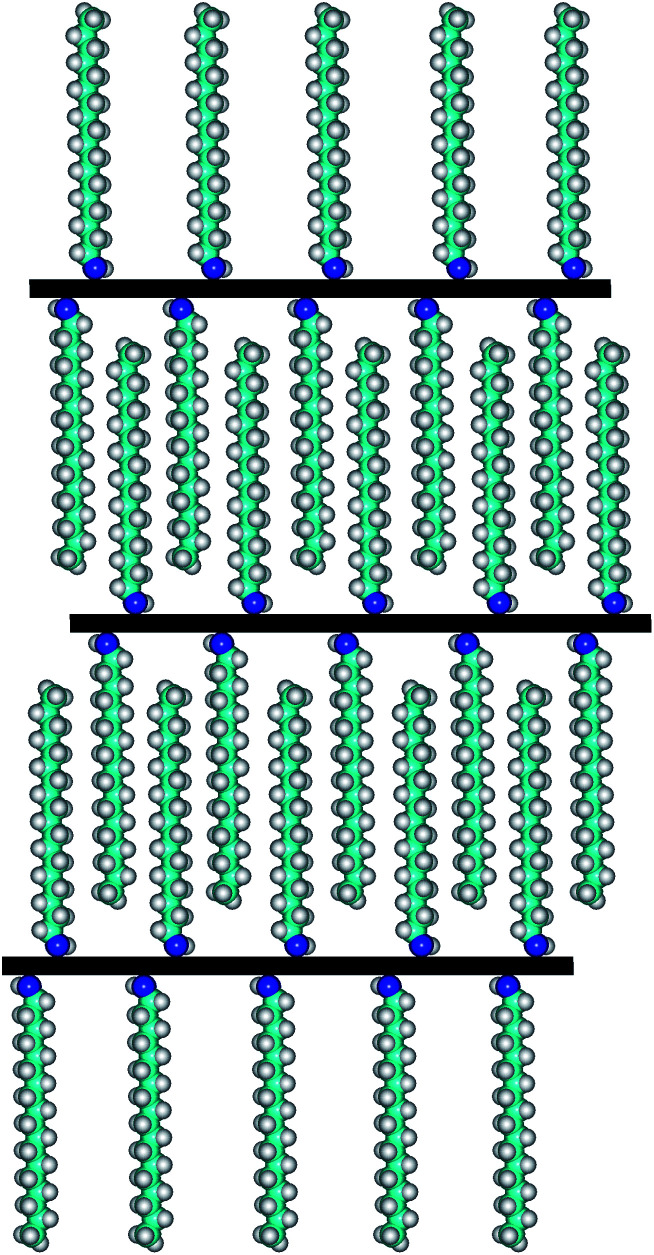
Schematic representation of the mechanism of GO nanosheet ordering in GOP-ODA.

For comparative morphology characterization of GOP mats before and after amine treatment, we used two microscopy techniques, namely SEM and AFM. The thickness of free-standing GOP before functionalization, determined from cross-section SEM images (Fig. 8a), was about 16 μm. An important detail is that the mats are formed by random stacking of individual wrinkled and folded sheets, as it was also observed by other research groups.^8,38^ After functionalization with amines, the average mat thickness was estimated as about 12, 12, 28 and 20 μm for GOP-AP, GOP-DAN, GOP-ODA and GOP-DAD: these changes can be appreciated from cross-section SEM images. Furthermore, Fig. 8d, g, j and m reveals that, unlike pristine GOP, amine-modified samples appear as relatively ordered layered structures, in which individual GO sheets are organized in a near-parallel fashion; this is especially clearly seen in the case of GOP-AP (Fig. 8d) and GOP-ODA (Fig. 8j). The underlying phenomenon for this ordering is the substitution of water molecules adsorbed on graphene oxide sheets (detectable as the reduced first weight loss in TGA curves) with amines, which, in turn, can produce two opposite effects, depending on functionalizing amine. In the case of GOP-AP and GOP-DAN, the aromatic ring systems attached to adjacent graphene oxide sheets interact through π–π stacking mechanism; at the same time, a relatively small length of AP and DAN molecules makes GO sheets approach each other, thus causing the reduction in mat thickness to about 12 μm. On the contrary, in the case of GOP-DAD and especially GOP-ODA, the linear hydrocarbon radicals are too long to fit the typical space between graphene oxide sheets, and thus make the interlayer distance increase. A possible specific mechanism for GO nanosheet ordering is exemplified for GOP-ODA in Fig. 9: it contemplates interdigitation of the hydrocarbon radicals linked to adjacent GO sheets. Octadecyl substituents are the longest ones among all amines employed in the present work, followed by somewhat shorter 12-aminododecyl radicals in the case of GOP-DAD. Correspondingly, mat thickness increases to about 28 and 20 μm for GOP-ODA and GOP-DAD, respectively. The possibility of tailoring the spacing in GO frameworks by varying the chain length of *n*-alkylamines, which was discussed by Mungse *et al.*,^30^ has much to do with the phenomenon observed in the present case. Apparently, it is also responsible for a very high value of Young's modulus obtained for GOP-ODA (256.19 GPa; Table 2), as well as for an increased thermal stability of GOP-ODA and GOP-DAD as compared to GOP found by TGA (Fig. 6).

From SEM images in the middle and right columns of Fig. 8 one can also note that the upper and the lower (with respect to the filter membrane) mat sides have slightly different morphologies. And what is more obvious is the appearance of grainy texture on GOP-ODA and GOP-DAD mat surfaces, which can be explained by a greater content of grafted organics, as found by TGA.

**Fig. 10:**
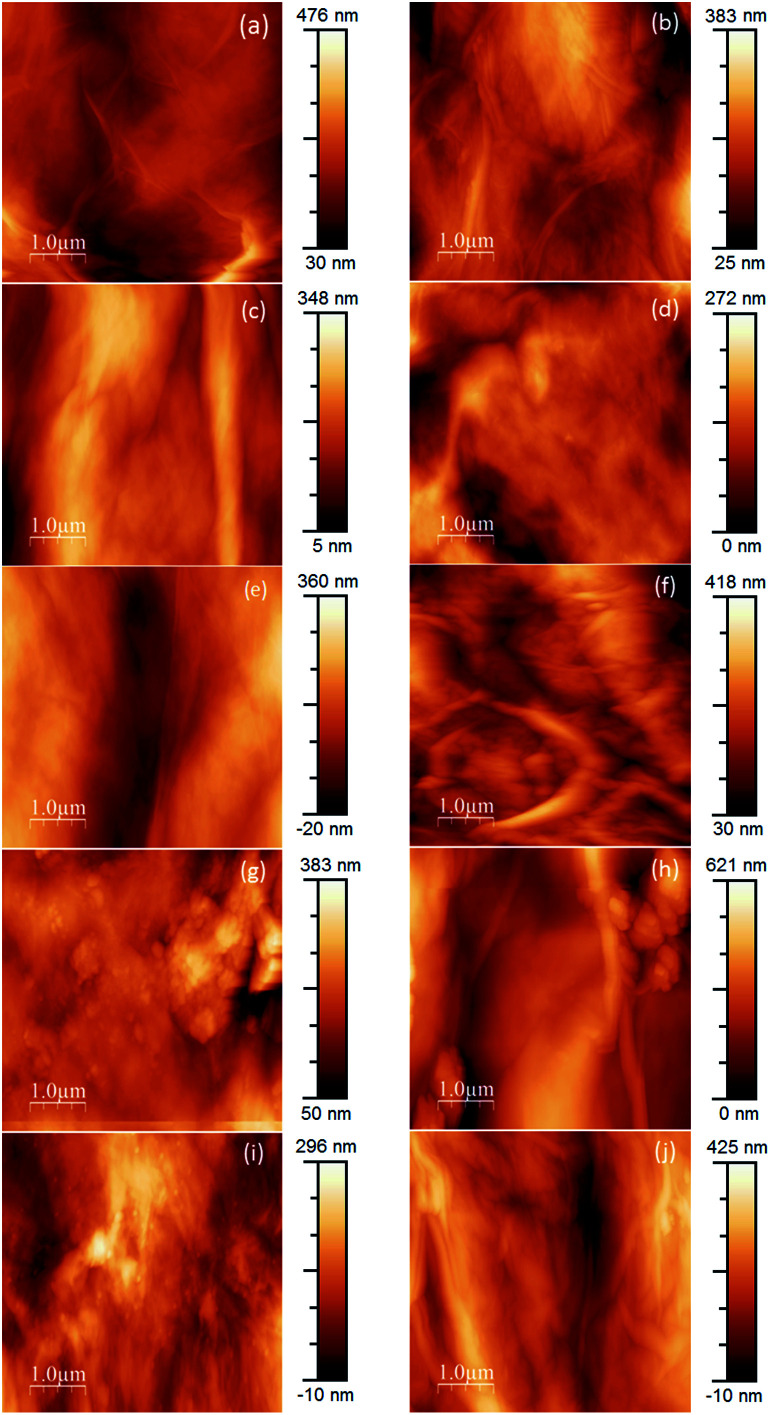
AFM images of pristine and amine-functionalized GOP samples. Pristine GOP (a and b), GOP-AP (c and d), GOP-DAN (e and f), GO-ODA (g and h) and GOP-DAD (i and j). The left column (a, c, e, g, i) shows the lower mat side which was in contact with the filter membrane during GOP fabrication; the right column (b, d, f, h, j), to the upper mat side.

As a whole, the AFM topography images obtained (Fig. 10) match the results of SEM observations. Overall, pristine GOP samples (Fig. 10a and b) have smoother surfaces, but always with wrinkled areas, which are characteristic for GO due to the presence of numerous oxidized defects.^39^ Amine functionalization generally increases the wrinkling, and in some cases mat surfaces have grainy appearance (Fig. 10f–i), which is more evident for GOP-ODA and GOP-DAD having a greater content of grafted organics.

**Fig. 11:**
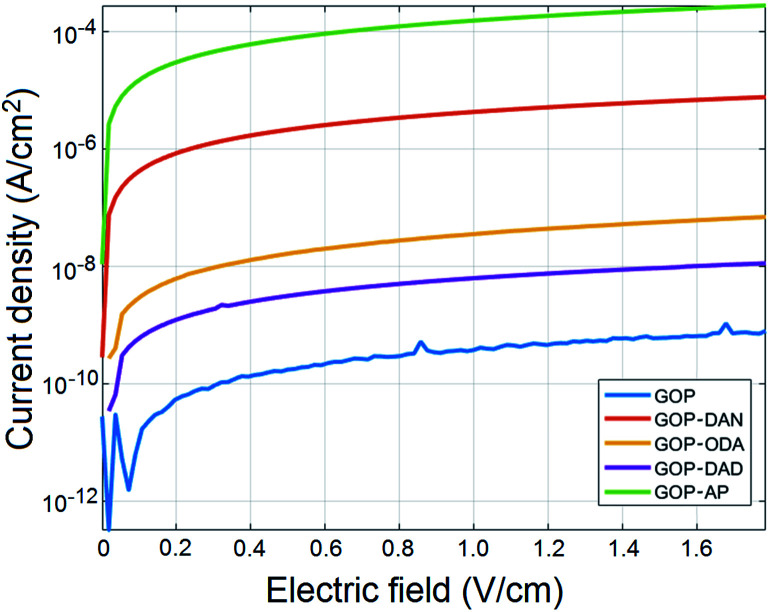
Current density–electric field measurements for pristine and functionalized GOP samples at room temperature and atmospheric pressure.

Previous studies by other research groups^10–12,40^ demonstrated that electrical conductivity of GOP can be enhanced by means of rather aggressive thermal and chemical treatments, exemplified by thermal annealing at temperatures of 300–700 °C and chemical reduction. Both approaches alter the chemical structure of GO by removing oxygen-containing functionalities and generating graphene structure, which in turn affects mechanical and structural properties of GOP and limits the range of its applications. From this point of view, it was interesting to study possible changes in electrical conductivity of amine-functionalized samples in comparison with pristine GOP. Fig. 11 shows the results of current density *vs.* electric field measurements for pristine and functionalized GOP mats under ambient conditions (room temperature and atmospheric pressure); due to dramatic variations in conductivity values found, the data are plotted on a logarithmic scale. In particular, the measurements revealed an increase by six orders of magnitude in GOP conductivity after functionalization with AP: as one can see from the Table 3, the conductivity of GOP-AP is as high as 1.55 ± 0.09 S cm^−1^ compared to the value of (4.56 ± 0.37) × 10^−6^ S cm^−1^ obtained for pristine GOP. For GOP-DAN, we also found a significant increase of four orders of magnitude, with a conductivity value of (4.30 ± 0.35) × 10^−2^ S cm^−1^. The functionalization with aliphatic amines ODA and DAD also improves electrical conductivity of GOP, by two orders of magnitude for ODA and by one order of magnitude for DAD, namely, to (3.67 ± 0.09) × 10^−4^ and (6.57 ± 0.68) × 10^−5^ S cm^−1^, respectively. The above increase in conductivity for amine-functionalized GOP can be explained by the formation of new connections between individual graphene oxide layers, through π–π stacking between aromatic rings of AP and DAN, and through hydrophobic interactions between aliphatic chains of ODA and DAD. The effect is much stronger in the case of aromatic amines, due to a higher mobility of π-electrons. In turn, the size of the fused aromatic system matters as well: AP, for which the highest conductivity was obtained, has a four-ring system, *versus* two fused rings in DAN. The increase in conductivity we measured is not as large as the one obtained for thermally annealed GOP by Vallés and coworkers.^12^ The difference between the two treatments is that annealing removes oxygenated groups from the lattice and restores the high electrical conductivity of pure graphene, whereas in our case one part of oxygenated functionalities (carboxylic and epoxy groups) chemically reacts with amine molecules, and the other part remains intact.

**Table 3:** Conductivity values obtained for pristine and functionalized GOP samples

Sample	Conductivity (S cm^−1^)
GOP	(4.56 ± 0.37) × 10^−6^
GOP-ODA	(3.67 ± 0.09) × 10^−4^
GOP-DAN	(4.30 ± 0.35) × 10^−2^
GOP-DAD	(6.57 ± 0.68) × 10^−5^
GOP-AP	(1.55 ± 0.09)

## 4. Conclusions

We demonstrated that the solvent-free functionalization with amines of different structure (aliphatic and aromatic, monofunctional and bifunctional) is a fast, efficient and nondestructive approach to systematically change GOP properties. The functionalization is carried out under moderate heating at 150–180 °C in vacuum, and proceeds through both amidation and epoxy ring opening reactions. According to TGA, the highest content of amine species is obtained in the case of GOP-ODA, followed by GOP-DAD, GOP-AP and GOP-DAN. The functionalization increases mechanical and thermal stability, as well as the electrical conductivity of GOP. The magnitude of each effect depends on the structure of amine employed, which allows for tuning a given GOP characteristic. Morphological characterization by SEM showed that, compared to pristine graphene oxide paper, amine-modified mats become relatively ordered layered structures, in which individual GO sheets are organized in a near-parallel fashion; this effect can be especially clearly seen in GOP-AP and GOP-ODA.

## Conflicts of interest

There are no conflicts to declare.

## Supplementary Material

RA-008-C8RA00986D-s001
