# Supplementary material for: One-step nondestructive functionalization of graphene oxide paper with amines
Source: RSC Adv. 2018 Apr 23;8(28):15253–65. doi: 10.1039/c8ra00986d (PMC9080034; doi:10.1039/c8ra00986d)
Supplement: RA-008-C8RA00986D-s001 [file RA-008-C8RA00986D-s001.pdf]

Electronic Supplementary Material (ESI) for RSC Advances.
This journal is © The Royal Society of Chemistry 2018

Electronic supplementary information

One-step nondestructive functionalization of graphene oxide paper with amines†

Natalia Alzate-Carvajal,[a] Diego A. Acevedo-Guzmán,[a] Victor Meza-Laguna,[b] Mario H. Farías,[c] Luis A. Pérez-Rey,[a] Edgar Abarca-Morales,[a] Victor A. García-Ramírez,[a] Vladimir A. Basiuk[b,]* and Elena V. Basiuk[a,]*

[a] *Centro de Ciencias Aplicadas y Desarrollo Tecnológico, Universidad Nacional Autónoma de México, Circuito Exterior C.U., Ciudad de México, 04510, Mexico*

[b] *Instituto de Ciencias Nucleares, Universidad Nacional Autónoma de México, Circuito Exterior C.U., Ciudad de México, 04510, Mexico*

[c] *Centro de Nanociencias y Nanotecnología, Universidad Nacional Autónoma de México, Apdo. Postal 14, Ensenada, 22800, Baja California, Mexico*

---

* Corresponding authors:

E-mail: basiuk@nucleares.unam.mx (V. A. Basiuk), elbg1111@gmail.com (E. V. Basiuk).

Measurements of water contact angles and surface free energy

The surface free energy was calculated by using the Owens–Wendt–Rabel–Kaelble (OWRK) model,[19] in which the surface free energy is divided into polar and disperse part. The relationship (commonly referred to as Young's equation) between the contact angle θ (Table S1), the surface tension of the liquid γ_l, the interfacial tension γ_{sl} between liquid and solid and the surface free energy γ_s of the solid is as follows:

$$\gamma_s = \gamma_{sl} + \gamma_l \cdot \cos(\theta) \quad (1)$$

The interfacial tension γ_{sl} is calculated based on the surface tensions γ_s and γ_l between the phases. These interactions are interpreted as the geometric mean of disperse part γ^D and polar part γ^P of the surface tension or surface free energy:

$$\gamma_{sl} = \gamma_s + \gamma_l - 2\left(\sqrt{\gamma_s^D \cdot \gamma_l^D} + \sqrt{\gamma_s^P + \gamma_l^P}\right) \quad (2)$$

At least two liquids (we used water and diiodomethane as reference; Table S2) with known disperse and polar parts of the surface tension are required to determine the surface free energy of the solid, wherein at least one of the liquids must have polar part >0.

If equations (1) and (2) are combined, the equation (3) called OWRK can be written as:

$$\sqrt{\gamma_s^D} + \sqrt{\gamma_s^P} \cdot \sqrt{\frac{\gamma_l^P}{\gamma_l^D}} = \frac{\gamma_l(1 + \cos[\theta))]}{2\sqrt{\gamma_l^D}} \quad (3)$$

Equation (3) can be represented in the linear form as

$$c + mx = y \quad (4)$$

where $c = \sqrt{\gamma_s^D}$, $m = \sqrt{\gamma_s^P}$ and $x = \sqrt{\frac{\gamma_l^P}{\gamma_l^D}}$

Table S1. Wettability results obtained for water and diiodomethane.

Sample	Water contact angle (°)	Diiodomethane contact angle (°)	Disperse (mN/m)	Polar (mN/m)
GOP	66.13	41.31	38.95	9.48
GOP-AP	84.10	41.64	38.78	2.40
GOP-DAN	87.96	42.04	38.51	1.48
GOP-ODA	102.77	68.07	23.96	0.59
GOP-DAD	90.07	36.35	41.40	0.78

Table S2. Surface tension of water and diiodomethane.

Solvent	Total surface tension (mN/m)	Disperse surface tension (mN/m)	Polar surface tension (mN/m)
Water	72.8	21.8	51.0
Diiodomethane	50.8	50.8	0.0
